# Facial *vs.* meridional coordination in gaseous Ni(ii)–hexacyclen complexes revealed with infrared ion spectroscopy†

**DOI:** 10.1039/d2cp03871d

**Published:** 2022-10-25

**Authors:** Musleh Uddin Munshi, Giel Berden, Jos Oomens

**Affiliations:** Department of Chemistry, Sogang University Seoul 04107 Korea musleh@sogang.ac.kr; Radboud University, Institute for Molecules and Materials, FELIX Laboratory, Toernooiveld 7 6525 ED Nijmegen The Netherlands j.oomens@science.ru.nl; University of Amsterdam, Science Park 904 1098XH Amsterdam The Netherlands

## Abstract

We report fingerprint infrared multiple-photon dissociation (IRMPD) spectra of the isolated gaseous hexa-coordinated complex of the macrocycle hexa-aza-18-crown-6 (hexacyclen, 1,4,7,10,13,16-hexaazacyclooctadecane, 18-azacrown-6) with Ni^2+^. The metal–ligand complexes are generated using electrospray ionization (ESI) and IR action spectra are recorded in a Fourier transform ion cyclotron resonance mass spectrometer (FTICR) MS coupled to the infrared free-electron laser FELIX. We investigate geometric structure of the complexes and in particular the chelation motif, by comparison with computed vibrational spectra, obtained using density functional theory (DFT) at the B3LYP/6-31++G(d,p) level. The quasi-octahedral chelation motif of the complex has been well documented in condensed-phase studies, and we focus here on the gas-phase structure, addressing in particular the question of a facial (*fac*) *versus* a *meridional* (*mer*) octahedral chelation geometry. Based on the good agreement between calculated linear IR spectra and experimental IRMPD spectra, we conclude that the gas-phase complex adopts a *mer* chelation geometry and we exclude significant contribution of the *fac* isomer, which is computed to lie about 10 kJ mol^−1^ higher in energy. We also address the possible presence of both meridional diastereomers and of higher energy conformers of meridional isomers. Finally, as expected for the d^8^ Ni^2+^-ion in an octahedral ligand environment, the IR spectrum also shows that the complexes are in a high-spin electron configuration.

## Abstract 2

The infrared multiple-photon dissociation (IRMPD) spectrum of the hexa-coordinated complex of hexacyclen (18-azacrown-6) with Ni^2+^ shows that the complex has a meridional – and not facial – chelation geometry in the gas phase.

## Graphical abstract

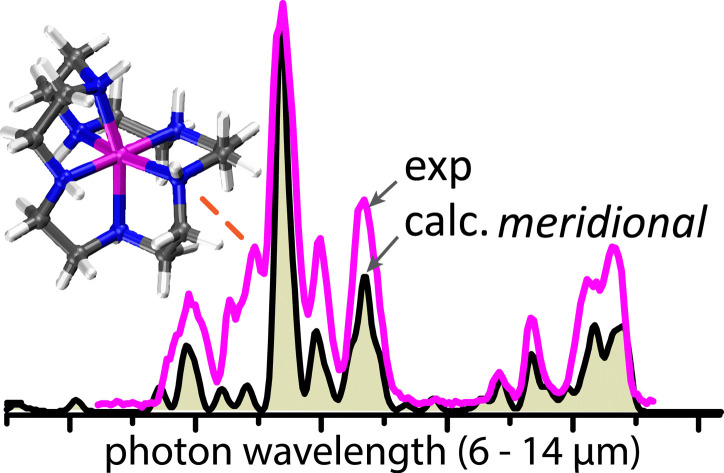

## 1. Introduction

Metal ions complexed with polydentate, macrocyclic ligands represent archetypal host-guest systems of high interest in the field of molecular recognition.^1^ Since their discovery,^2^ complexation of crown ethers with metal ions has attracted much attention, because of their relevance in chemical, biochemical, industrial and medical applications, which include for instance extraction and sensing of substances from solutions (including anions),^3–5^ purification of water^6,7^*via* supramolecular complexation technologies,^8,9^ removal of toxic radio-isotopes from nuclear waste,^10–12^ transport of radioactive cytolytic reagents to tumors,^13^ applications in the design of advanced analytical methods^12^ and metal ion catalysis and organic synthesis.^14^ Studies of the complexation of macrocycles with metal cations cover nearly the entire periodic table from alkali,^3,15–17^ alkaline earth,^18,19^ and first row transition metals^20^ to the lanthanides and actinides.^21,22^ A major thrust for experimental and theoretical studies has been the investigation of model macrocyclic-metal complexation mimicking many relevant biological systems (*e.g.*, the porphyrin ring in haem protein, the cyclic complex in chlorophyll, and the Corrine ring in vitamin B_12_) with the aim to decipher their structure and function.^14,23,24^

Apart from nuclear magnetic resonance (NMR) and infrared (IR) spectroscopy, circular dichroism (CD) has been extensively used for their characterization in solution, exploiting the optical isomerism of these coordination complexes.^25^ Considerable effort was also spent on their theoretical modelling^25–28^ to rationalize the observed spectral features, especially regarding the intrinsic d–d transitions in chiral transition–metal complexes. Theoretical advances depend strongly on the availability of experimental data on suitable model systems with known absolute configurations, preferably in complete isolation as interactions with the solvent and temperature effects may blur the phenomena of interest.^29,30^

Ion storage mass spectrometry forms an excellent platform for the study of charged gaseous metal–ligand complexes in complete isolation, removing any influence of solvent interactions. Recently, Rodgers and co-workers investigated complexation of alkali metal ions with hexacyclen to determine bond dissociation energies (BDE) using threshold collision-induced dissociation (CID) MS. Discrepancies were noted in the BDE between experiment and theory due to the lack of isomer selectivity, and the possible presence of higher-energy conformers in the experiment. Later an explanation was provided theoretically as to how the higher energy conformers could be formed.^15,16^ Martinez-Haya and coworkers^18,19^ investigated complexation of alkaline-earth metal cations (Mg^2+^, Ca^2+^, Sr^2+^, and Ba^2+^) with 18-crown-6 ether (the O analog of hexacyclen) using IRMPD action spectroscopy. Their experiment revealed that these metal ions formed similar but more tightly bound complexes than alkali metals. Both types of metal cations formed hexa-coordinated complexes, where the metal ion remains encapsulated inside the folded macrocyclic ligand. The alkali and alkaline-earth metal ions form equatorial chelation complexes with the 18-crown ligand, clearly distinguishable from an octahedral geometry in the IRMPD spectroscopy and DFT.

**Fig. 1:**
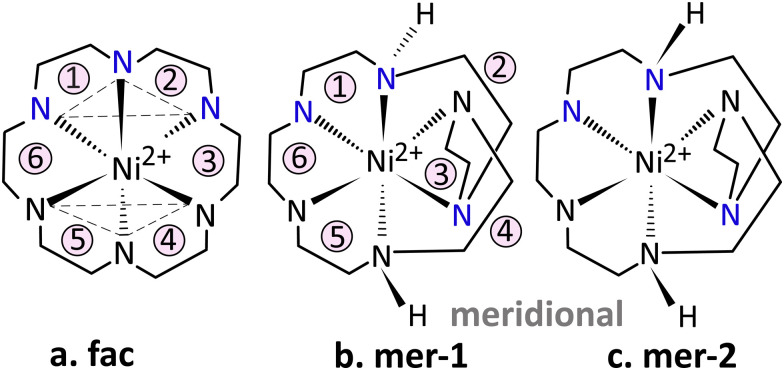
Geometric isomers of [Ni(hexacyclen)]^2+^: (a) *fac* (*Δ*,*Λ-δλδλδλ*), (b) *mer*-1 (*Λ*,*Λ-δλλλδλ*) and (c) *mer*-2 (*Λ*,*Λ-λδλλδλ*). Stereoisomers have facial (*fac*) and meridional (*mer*-1, *mer*-2) Ni–N linkages.

Complexation of divalent first-row transition metals with hexacyclen forms octahedrally coordinated complexes. Computational investigation using DFT further predicts that these ions prefer the *meridional* isomeric form over the *facial* isomer (Fig. 1) and that they may co-exist.^20^ It was also concluded that H–H interactions in large metal–ligand complexes could be interpreted as steric hindrance and thus destabilizing in nature. The prediction on the preference for *meridional* or *facial* isomers generally agreed well with the available isomer distribution determined experimentally. For instance, complexation of Co^3+^ with hexacyclen yielded approximately 99% *meridional* and about 1% *facial* isomer in solution.^31^ Despite these studies, gas-phase data is scarce, especially for complexation of transition metal ions in an octahedral ligand environment. It often remains challenging to correctly predict the geometrical and spectroscopic properties of transition metal–ligand complexes,^32–34^ because of the near-degeneracy among the partially filled d-shells.^35^ Ni^2+^(d^8^) in an octahedral ligand environment falls within this paradigm.

Two types of geometrical isomers exist for [Ni(hexacyclen)]^2+^, *facial* (*fac*) and *meridional* (*mer*). In the *fac* isomer, all chelate N–C–C–N–C–C–N linkages are facial, whereas in the *mer* isomer, two of the linkages are *meridional*.^25,31,36,37^ The *mer* isomer can exist as two stereoisomers that occur as racemates. These stereoisomers are further sub-divided into two diastereomers (*mer*-1 and *mer*-2) due to an alternative disposition of the H-atoms on the meridionally coordinated nitrogens. This reduces the molecular symmetry to *C*_2_ as in the bis(diethylenetriamine)cobalt(iii) ion^38^ while chelate ring conformations are *λδλλδλ* (*mer*-1) and *δλλλδλ* (*mer*-2) in the order of chelate rings depicted in Fig. 1. Similarly, *fac* has *δλδλδλ* conformation.

In this work, we present the IR spectrum of the gaseous, isolated [Ni(hexacyclen)]^2+^ complex to characterize its electronic and geometric structure. We examine the complex employing IRMPD spectroscopy^39–41^ in an FTICR MS^42,43^ coupled to the wavelength tunable infrared free electron laser FELIX. DFT computed vibrational spectra are compared with the experimental action spectra to extract structural information from the experimental spectra.

## 2. Methods

### 2.1. IRMPD action spectroscopy

Experiments were performed in a home-built FTICR MS.^42,43^ The dication of interest, [Ni(hexacyclen)]^2+^ at *m*/*z* 158, was generated by electrospray ionization (ESI) starting from a solution containing equimolar (0.5 mM) Ni(NO_3_)_2_ salt and hexacyclen in 1 : 1 MeOH : H_2_O. Fingerprint IRMPD spectra were recorded from 700 to 1550 cm^−1^ using the tunable infrared radiation from the FELIX free electron laser (FEL).^44^ FELIX^45^ was operating at a repetition rate of 10 Hz producing 6–10 μs long macropulses with energies up to 200 mJ per pulse.

In the FTICR MS, ions were generated using a Z-Spray ESI source (Micromass, UK). Ions were accumulated for about 6 seconds in a linear hexapole trap and then injected into the ICR cell *via* a quadrupole deflector and an rf octopole ion guide. Target ions were isolated using a stored waveform inverse Fourier transform (SWIFT) excitation pulse and subsequently irradiated with 50 FEL macro pulses to record the IRMPD spectra. IRMPD spectra were obtained by monitoring the wavelength-dependent photo-fragmentation, using a 5 cm^−1^ step size and two summed mass spectra at each wavelength. Two major fragments (*m*/*z* 111 and 97) were observed; at the dominant bands only *m*/*z* 97 was observed presumably due to secondary fragmentation of *m*/*z* 111 into *m*/*z* 97.

The mass-selected ions are vibrationally excited whenever the laser frequency is in resonance with one of the normal mode frequencies of the ions. Multiple photons were absorbed at these frequencies and statistical redistribution of the absorbed energy promoted the increase of the internal energy of the ions *via* intramolecular vibrational redistribution (IVR).^46,47^ Once the energy exceeds the lowest energy dissociation limit in the molecule, the ion undergoes fragmentation. IR spectra were generated by plotting the fragmentation yield (eqn (1)) or the depletion (eqn (2)) of the precursor ions as a function of laser frequency.^48^1
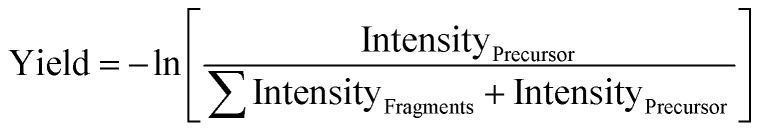
2
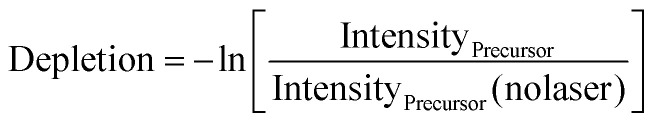


The fragmentation yield and precursor ion depletion were linearly corrected for frequency-dependent variations in the laser pulse energy and a grating spectrometer was used to calibrate the IR frequencies.

### 2.2. Computational modelling

Gas-phase geometries were optimized at the density functional level of theory (DFT) using the hybrid B3LYP^49,50^ functional with the 6-31++G(d,p) basis set. Using the polarizable continuum model (PCM), geometries were also optimized in water. At each minimum located, single-point (SP) energy calculations were performed at the MP2/6-311+G(d,p) level to better assess the relative stabilities of the isomers. All calculations employed the Gaussian16^51^ computational program package.

Harmonic vibrational frequencies were calculated for the optimized geometries. No imaginary frequencies were found, confirming that the stationary points were true minima on the potential energy hypersurface. Computed frequencies were convoluted using a 20 cm^−1^ full-width at half-maximum (FWHM) Gaussian line shape function, such as to match the observed bandwidth in the IRMPD spectra. Computed (harmonic) IR frequencies were scaled^52,53^ by a factor of 0.97 to compensate for the anharmonicity and basis set incompleteness.

Additionally, MP2/6-31++G(d,p) and M06/TZ2P levels of theory were employed to possibly refine computed relative intensities, IR band positions and to interrogate deviations from experiment. Despite the similar optimized geometries, predicted IR spectra appeared to provide poorer matches with experiment than those obtained at the B3LYP/6-31++G(d,p) level (Fig. S1 and S2, ESI†) and hence these will not be discussed further. Ni(ii) has a d^8^ electron configuration, favouring a high-spin (triplet) state in an approximately octahedral coordination environment, rather independent of the magnitude of the ligand field splitting. Indeed, the mismatch between the experimental and calculated IR spectra for low-spin (singlet) complexes shown in Fig. S3, ESI† leads us to consider only high-spin complexes.

## 3. Results and discussion

### 3.1. Computed geometries of the *fac* and *mer* isomers

**Fig. 2:**
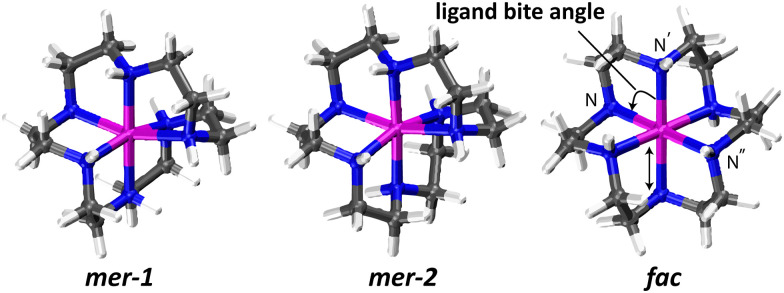
B3LYP/6-31++g(d,p) optimized geometries of [Ni(hexacyclen)]^2+^ in their high-spin states. Ni–N bond length and ligand bite angle (N–Ni–N) listed in Table 2 are indicated.

Fig. 2 shows the optimized *fac* and *mer* isomers with the latter in its two diastereomeric forms. Key structural parameters are summarized in Table 1. Relative energies for the different isomers (each in their lowest-energy conformational form, *vide infra*) are listed in Table 2.

**Table 1:** Optimized structural parameters of the isomers of [Ni(hexacyclen)]^2+^ in the gas phase. Atom labels are shown in Fig. 2

Bond length	*mer*-1	*mer*-2	*fac*
d(Ni–N) (Å)	2.187	2.188	2.198
Bond angle (°)			
N–Ni–N′	80.8	80.6	81.1
N–Ni–N′′	165.9	165.7	180.0

**Table 2:** Computed relative free energies in kJ mol^−1^ of *fac* and *mer* isomers in the gas phase and in water. Single point (SP) energies are calculated at the MP2/6-311+G(d,p) level using the B3LYP/6-31++G(d,p) geometries

	B3LYP/6-31++G(d,p)		SP-MP2/6-311+G(d,p)		M06/TZ2P	B3LYP/aug-cc-PVTZ	MP2/6-31++G(d,p)
Isomers	Water	Gas	Water	Gas	Gas	Gas	Gas
*mer*-2	1.5	0	1.0	1.3	0.02	0.2	3.2
*mer*-1	0	0.4	0	0	0	0	0
*fac*	10.7	9.7	3.6	3.2	9.5	7.8	7.8

The complexes show pseudo-octahedral coordination of the six nitrogen atoms to the central nickel ion. The *fac* isomer retains a highly symmetrical (*D*_3d_) geometry while the *meridional* isomers have approximate *C*_2_ symmetry. In contrast to *mer*, *fac* shows highly regular Ni–N distances (2.198 Å) and ligand bite angles (∠N–Ni–N′ = 81°). The four equatorial N-atoms remain in the plane (∠NNNN = 0°) perfectly, while keeping the metal at the center. The two axial N-atoms remain linear (∠N–Ni–N′′ = 180°) with the metal center without being perfectly orthogonal to the equatorial plane.

It is noteworthy that the metal centered Ni–N coordination sphere of the two *meridional* isomers match within 0.07 Å RMSD in atom positions. The average Ni–N distances of *meridional* isomers and their ligand bite angles (∠N–Ni–N′ = 80.6° for *mer*-2 and 80.8° for *mer*-1) are slightly decreased relative to *fac*. The subtle structural changes between *mer* and *fac* are reflected in their calculated IR spectra; as we shall see below, the IR spectra are sufficiently distinct in the fingerprint range (400–1800 cm^−1^) to identify the experimental ion population as (predominantly) *mer*. In comparison to X-ray crystallographic data,^54^ calculated average Ni–N distances for *meridional* isomers are in close agreement, being only 0.1 Å larger than experiment (2.08 Å). The same holds for the *fac* isomer where the calculated Ni–N bond length of 2.198 is slightly larger than the experimental value of 2.155 (± 0.001 Å).

At the DFT level of theory, the *fac* isomer is computed to lie approximately 8–10 kJ mol^−1^ higher in energy than both of the *mer* isomers, which are virtually iso-energetic (see Table 2). All DFT levels of theory employed confirm this general trend, although a triple-zeta basis set slightly reduces the *mer–fac* energy gap. The MP2 single-point calculation predicts a substantially smaller gap, but a full MP2 geometry optimization (with a smaller basis set) predicts a gap close to the DFT values. The increased stability of the *mer*-isomers relative to *fac* is in agreement with previous computational investigations and has been attributed to the closer proximity of the methylene units in the latter, increasing the steric hindrance.^20,31^ The energetics of the system, including alternative conformers, are further addressed below.

### 3.2. IRMPD spectra of [Ni(hexacyclen)]^2+^

**Fig. 3:**
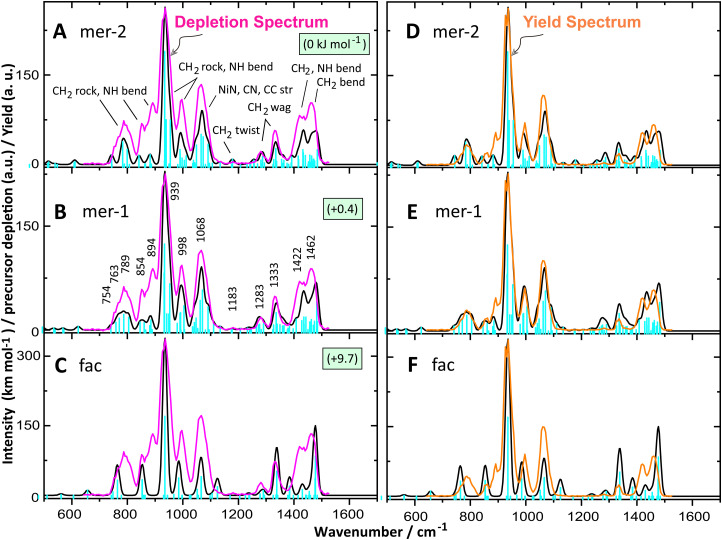
IRMPD spectrum of [Ni(hexacyclen)]^2+^ as depletion spectrum (magenta trace) overlaid with the calculated IR spectra (black) of *mer*-2 (A), *mer*-1 (B) and *fac* (C) at the B3LYP/6-31++g(d,p) level in their high-spin state. Relative Gibbs free energies are shown. Approximate vibrational mode characters are indicated in panel A. The panels to the right show the corresponding IR photodissociation yield spectrum (orange trace) overlaid with the same calculated IR spectra of *mer*-2 (D), *mer*-1 (E) and *fac* (F).

Fig. 3 shows the experimental IRMPD spectrum of the Ni(ii)–hexacyclen complex obtained in the FTICR MS represented as a precursor ion depletion spectrum (left panels) and as an IR photodissociation yield spectrum (right panels). The IR spectra in depletion and in yield are qualitatively consistent, although relative band intensities are slightly different. We attribute this discrepancy to (1) weak fragment ion signals that disappear in the noise on weak absorption bands in the yield spectrum of eqn (1) and (2) fluctuations in the ion signal from the ESI source, influencing the depletion spectrum through the denominator of eqn (2).^48^ Since the depletion spectrum contains more IR features than the yield spectrum, the interpretation of the spectrum by comparison with theoretical spectra as presented below is based on the spectrum observed in depletion.

### 3.3. IR spectral comparison with theory

Fig. 3 shows the comparison between the experimental IR spectrum of [Ni(hexacyclen)]^2+^ and the calculated linear IR spectra of potential isomers. *mer*-2 and *mer*-1 are virtually iso-energetic and correspond to the minimum energy structure. Computed spectra of these isomers are apparently similar, although slight differences are noticed in terms of band positions and relative intensities. Similar IR spectra are expected since their geometries differ only in the relative orientation of the NH hydrogens.

Band assignments in the 1200–1500 cm^−1^ range include the CH_2_ twisting, NH bending and CH_2_ bending vibrations. These vibrational normal modes are characteristic of the hexacyclen ligand. Similar bands were previously observed in the gas-phase spectra of the analogous macrocyclic tetradentate ligand cyclam complexed with copper(ii/i) and nickel(ii/i).^55,56^ Predicted bands for the minimum energy isomer match the observed bands in terms of relative intensities and band positions, although the observed bands are slightly red-shifted in the 1400–1500 cm^−1^ range. Computed IR bands in the range 600–1200 cm^−1^ are also very similar for both *meridional* isomers and these predicted IR bands also match the experimental spectrum closely. This range is populated by the CH_2_ rock, NH bend, CH_2_ twist and NiN, CC, CN stretching vibrations. The most dominant IR band is observed at 939 cm^−1^, matching with the predicted band at 934 cm^−1^ (for both *mer*-1 and *mer*-2) convolved with two features at slightly higher frequency, placing the band center exactly on top of the experimental feature. To the blue of the dominant band, two broadened bands centered at 998 and 1068 cm^−1^ are observed. Theory predicts these bands accurately for both *meridional* isomers, 992 and 1070 cm^−1^ for *mer*-1 and 997 and 1067 cm^−1^ for *mer*-2. A low intensity band is observed at 1183 cm^−1^ that is predicted at 1179 cm^−1^ for *mer*-2 for the characteristic CH_2_ twisting vibration. In the low frequency region 700–900 cm^−1^, three band maxima are observed at 789, 854 and 894 cm^−1^. These IR bands are also well-predicted at 787, 860, 884 cm^−1^ for *mer*-1 and 784, 842, 882 cm^−1^ for *mer*-2, respectively. Relative intensities of the observed bands are higher compared to the prediction. This may be due to artificial intensity enhancement due to the adjacent strong band at 939 cm^−1^, a known artifact in IRMPD spectra.^57,58^

The *fac* isomer lies about 10 kJ mol^−1^ higher in energy than the *mer* isomer. One notices that the predicted spectrum for this isomer does not nearly match as well with experiment. Nonetheless, many of the predicted IR bands are accommodated beneath the experimentally observed envelop (Fig. 3c and f), which does not allow us to completely rule out any contribution of the *fac* isomer to the ion population. Most of the calculated IR features are indeed similar to the *mer* isomers, with small apparent differences. For instance, in the range 1200–1500 cm^−1^, observed IR bands at 1283 and 1333 cm^−1^ are predicted to be at 1290 and 1339 cm^−1^ and their relative band shapes match the experiment. The relative intensity of the band at 1339 cm^−1^ is calculated to be higher in comparison to that of *mer* isomers. In contrast, the relative intensity of the IR band at 1430 cm^−1^ is much lower in *fac* than in the *mer* isomers.

In the region 600–1200 cm^−1^, six discrete IR bands are predicted for this *fac* isomer. Calculated dominant IR bands at 935, 985 and 1066 cm^−1^ overlap with observed bands at 939, 998 and 1068 cm^−1^. A relatively low intensity IR band is predicted at 1125 cm^−1^, which falls perhaps in the tail of the observed band at 1068 cm^−1^. In the 700–900 cm^−1^ range, two bands are predicted falling within the experimental envelope, although in this range the *mer* isomers clearly provide a better match to the spectrum overall, underpinning their predominant (if not exclusive) presence in the total ion population.

### 3.4. Isomer population

To estimate the isomer population based on their relative thermochemistry in the gas phase (Table 2), isomers are optimized also in aqueous solution, considering that the gas-phase population may depend on the population in the ESI-solution. Despite a tiny reversal of relative energies of *mer*-1 and *mer*-2, both *meridional* isomers can be considered isoenergetic with a 1.5 kJ mol^−1^ computed energy difference. Furthermore, *fac* remains higher in energy by 10.7 kJ mol^−1^ in water similar to the gas-phase energy gap. Hence, *meridional* isomers are favored equally in water as well as in the gas phase. Taking a room-temperature Boltzmann distribution, these relative energies suggest that there is 98% *meridional* and 2% *facial* present in the gas phase. These estimations are consistent with the experimental isomer population of the analogous [Co(hexacyclen)]^3+^ investigated using chromatography followed by nuclear magnetic resonance (NMR)^31^ and circular dichroism (CD) spectroscopy^59^ in solution. Taking the MP2/6-311+G(d,p) single point energies instead of the B3LYP/6-31++G(d,p) values, we find that the energy difference between *mer* and *fac* is reduced to about 3.5 kJ mol^−1^, both in water and in the gas phase. This would suggest a 25% contribution of *fac* to the Boltzmann population, which appears incompatible with the observed spectrum in Fig. 3.

### 3.5. Conformational flexibility

The 18c6-azacrown macrocycle contains six ethylene (–CH_2_–CH_2_–) units that are linked by six nitrogen donor atoms. These constitute rotatable single bonds that facilitate the formation of various conformers of each of the hexa-coordinated metal–ligand isomers. For each isomer, six conformers of higher energy can be generated in addition to the lowest-energy conformer. For the *mer*-1 isomer identified in the previous section, relative energies of these alternative conformations are up to 25 kJ mol^−1^ higher than the lowest-energy conformer. Their geometries are shown in Fig. S4 (ESI†), merged with the lowest-energy conformer to better visualize the structural differences. Transition-state calculations were performed connecting the different conformers revealing relatively low barriers of about 20–40 kJ mol^−1^ (see Fig. S5, ESI†).

**Fig. 4:**
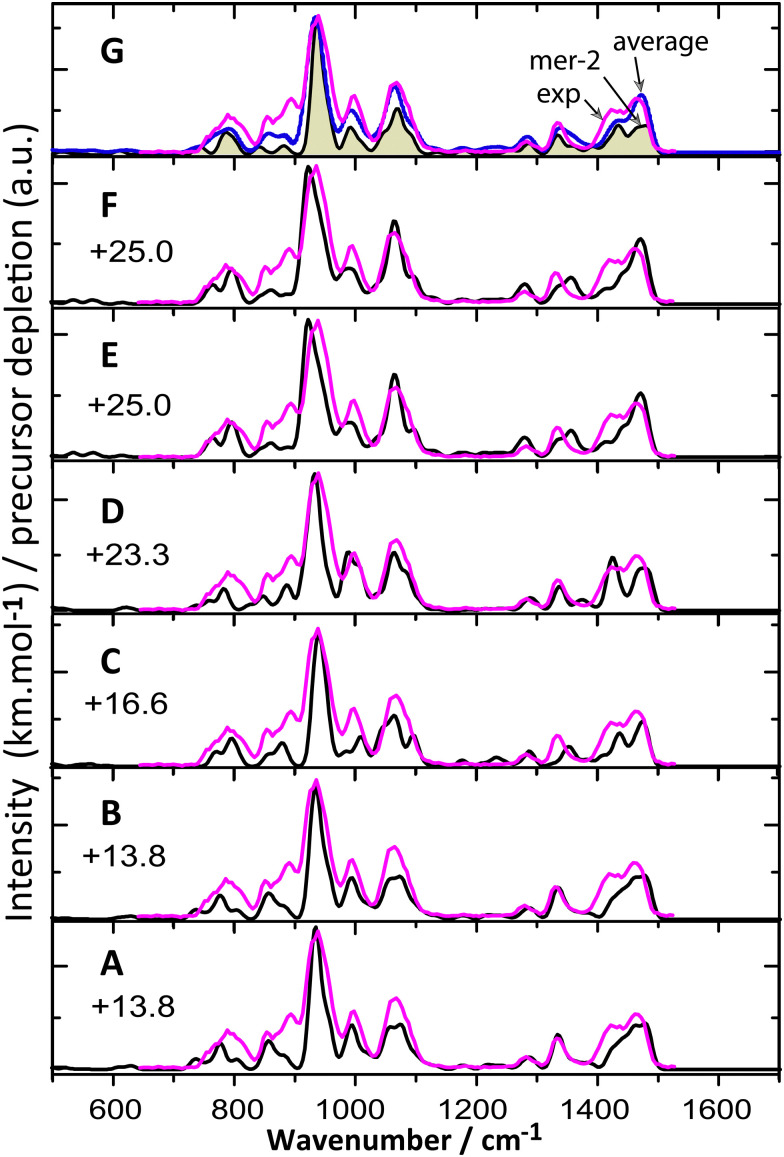
IRMPD spectrum of [Ni(hexacyclen)]^2+^ compared with the calculated IR spectra (black) of the higher energy conformers (A–F) of *mer*-1 in their high-spin state at the B3LYP/6-31++G(d,p) level. Gibbs free energies (kJ mol^−1^) are shown with respect to the global minimum *mer*-1 conformer. An average spectrum of six conformers is compared with the experiment in panel G. Optimized geometries for these conformers are presented in Fig. S4 (ESI†).

Fig. 4 shows a comparison between their predicted IR spectra and the measured IRMPD spectrum. As expected, the computed IR spectra of the different conformers are very similar, although one can also note subtle differences. For the highest-energy conformers (panel E, F), a slight red-shift is observed for the dominant IR band at 921 cm^−1^, which is observed 939 cm^−1^. In addition, slight deviations are also observed between the conformers around 1350 cm^−1^ in terms of the convoluted band shape. Overall, spectral differences are too small to reliably assess their presence or absence in the experimental population; their computed relative energies argues against their population. Fig. 4G displays an arithmetic average of the IR spectra of these six conformers, which matches well with experiment.

## 4. Conclusions

We have measured the IRMPD spectrum of *m*/*z* selected [Ni(hexacyclen)]^2+^ in the gas phase using the FELIX free-electron laser coupled to an FTICR MS to establish the chelation structure of the complex. Ni^2+^ is chelated by the macrocycle in an approximate octahedral configuration with a high-spin electronic configuration. Moreover, DFT calculations at the B3LYP level clearly assign the *meridional* isomeric form to the experimental ion population and exclude a significant contribution from the alternative *facial* isomer. Calculated thermochemistry agrees with our assignment in the sense that the *meridional* isomers are lower in energy than the *facial* by about 10 kJ mol^−1^ and this difference is estimated to be roughly the same in the ESI solvent. The predicted energetic difference between the two *meridional* diastereomers is negligibly small and their computed IR spectra are practically identical; we assume the co-existence of both species. The match between experimental and theoretical spectra appears to slightly improve when including higher-energy conformers of the *meridional* isomer. Nonetheless, the spectra of the different conformers are too similar to reliably assess the presence or absence of these conformers, and moreover, the alternative conformers are predicted to be significantly higher in energy (14–25 kJ mol^−1^), so that their actual presence in the ion population is unlikely.

## Conflicts of interest

There are no conflicts of interest to declare.

## Supplementary Material

CP-024-D2CP03871D-s001
