# Supplementary material for: Facial vs. meridional coordination in gaseous Ni(ii)–hexacyclen complexes revealed with infrared ion spectroscopy
Source: Phys Chem Chem Phys. 2022 Oct 25;24(43):26890–7. doi: 10.1039/d2cp03871d (PMC9644429; doi:10.1039/d2cp03871d)
Supplement: CP-024-D2CP03871D-s001 [file CP-024-D2CP03871D-s001.pdf]

Electronic Supplementary Material (ESI) for Physical Chemistry Chemical Physics.
This journal is © the Owner Societies 2022

## Supplementary info

# Facial vs. meridional coordination in gaseous Ni(II)-hexacyclen complexes revealed with infrared ion spectroscopy

**Musleh Uddin Munshi[a*], Giel Berden[b], Jos Oomens[b, c*]**

[a]Department of Chemistry, Sogang University, Seoul 04107, Korea, e-mail: musleh@sogang.ac.kr

[b]Radboud University, Institute for Molecules and Materials, FELIX Laboratory, Toernooiveld 7c, 6525 ED Nijmegen, The Netherlands

[c]University of Amsterdam, Science Park 904, 1098XH Amsterdam, The Netherlands, e-mail: j.oomens@science.ru.nl

# Supplementary Information

## Computational modeling: MP2 and M06 functional

MP2/6-31++G(d,p) level calculations were performed using the Gaussian16 revision C 01[1] computational program package. Computed harmonic IR frequencies are scaled by a factor of 0.95.[2,3] MP2/6-31++G(d,p) level computed IR spectra shown in Fig S1 look similar to those obtained at the B3LYP/6-31++G(d,p) level in terms of band intensities, but the IR band positions are not predicted as correctly as at B3LYP/6-31++G(d,p) in comparison with the experiment. We note that optimized geometries at both levels of theory are similar, which is represented as RMSD (in Å) in Fig S1.

In addition, the Amsterdam Density Functional (ADF) program package[3–5] was used, where the meta functional M06 as recently developed by the Truhlar group[2] was employed with uncontracted Slater type orbitals (STOs) of triple-ζ quality, including two sets of polarization functions (TZ2P[4]). Computed harmonic IR frequencies are scaled by a factor of 0.98. Computed IR spectra at the M06/TZ2P level (Figure S2) are not as good as the B3LYP/6-31++G(d,p) level, despite the fact that optimized geometries are in good agreement between both levels of theory. Calculated RMSDs for the best fit between B3LYP and M06 levels for all three isomers are on the order of 0.03 Å (Figure S2), indicating that the geometries are nearly identical (Chemcraft 1.8 implementation of the RMSD calculation is used).

# Supplementary info

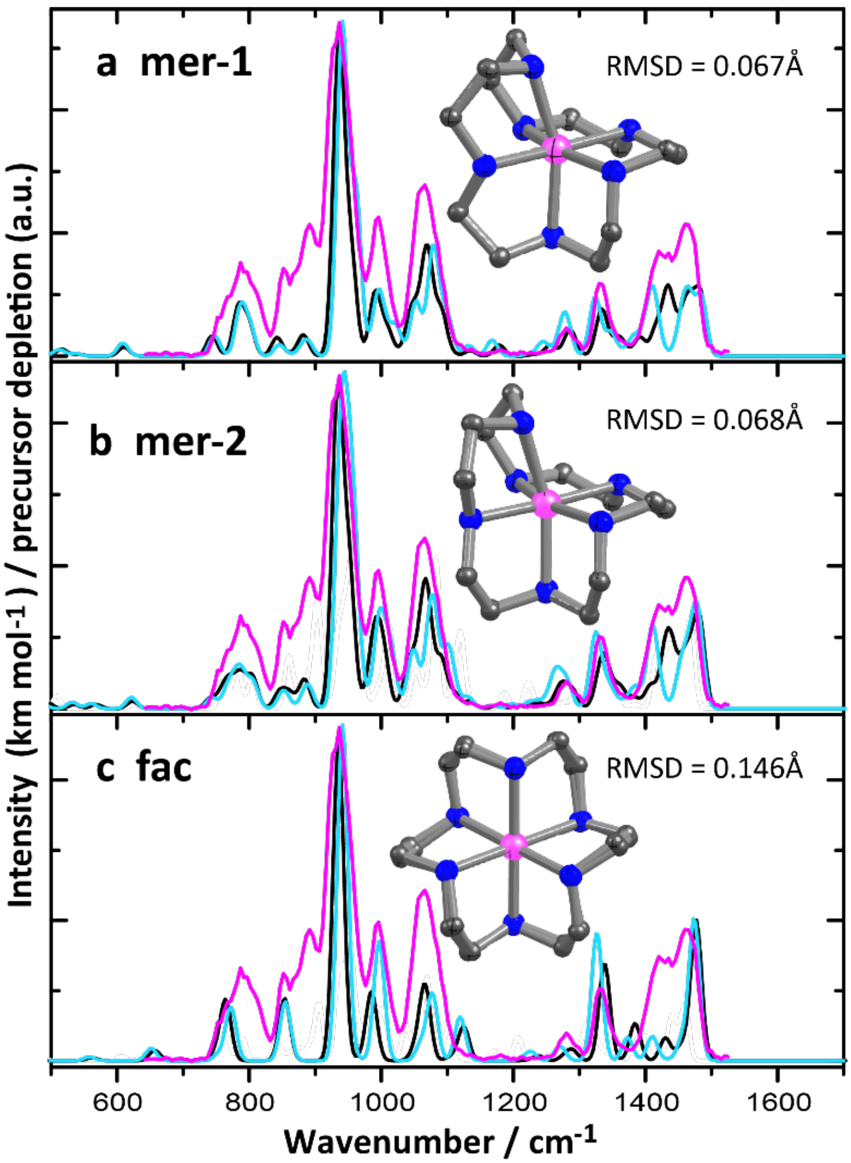


**Figure S1** IRMPD spectrum of $[Ni(hexacyclen)]^{2+}$ (magenta trace) overlaid with the calculated IR spectra of the isomers a) *mer*-1, b) *mer*-2, and c) *fac* in their high-spin state at the MP2/6-31++G(d,p) (cyan) and B3LYP/6-31++G(d,p) (black) levels. The geometrical similarity between both theoretical levels is indicated as a root mean square deviation (RMSD) of the atom positions in Å.

## Supplementary info

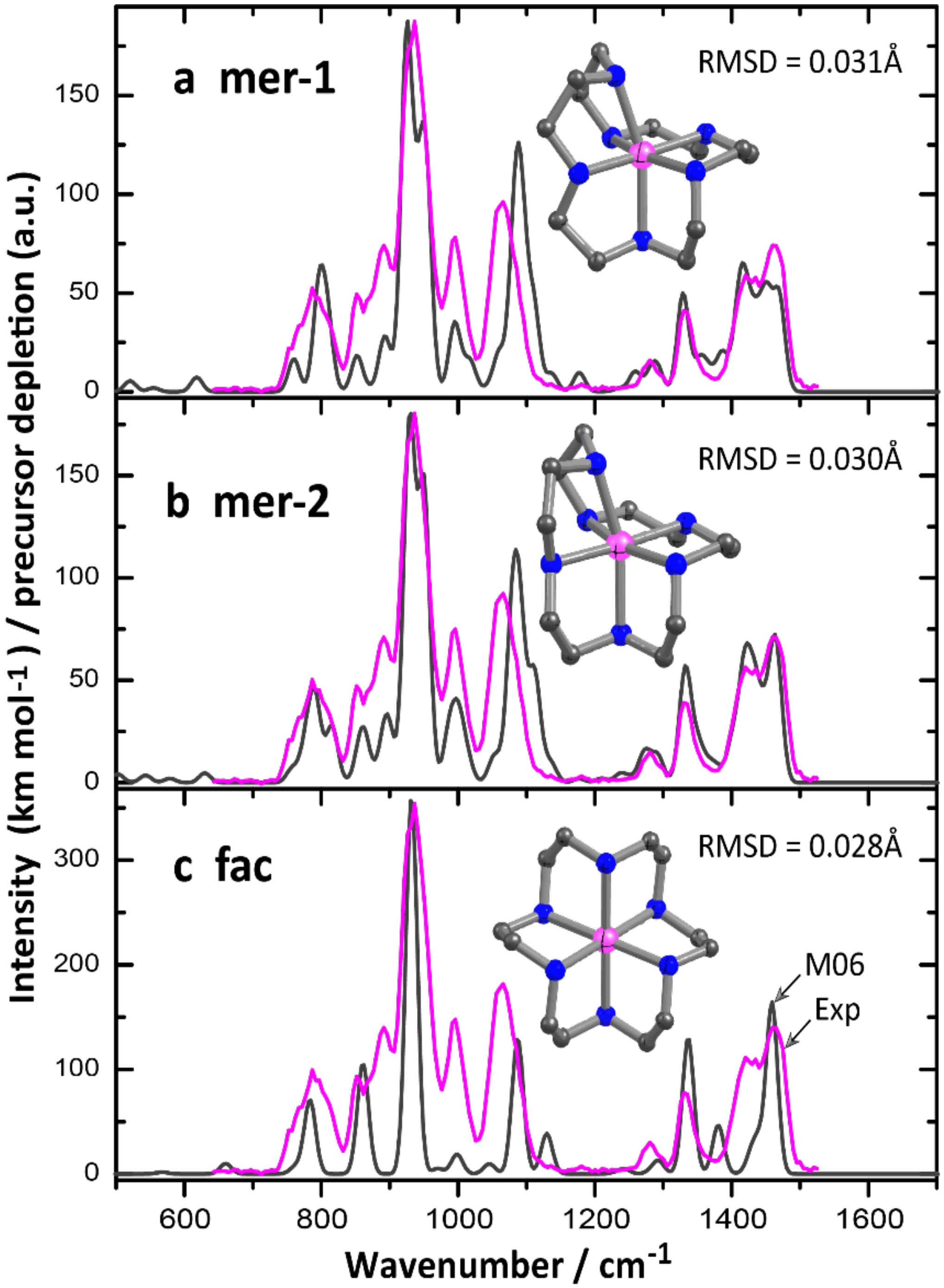


**Figure S2** IRMPD spectrum of $[Ni(hexacyclen)]^{2+}$ (magenta trace) overlaid with the calculated IR spectra (gray) of the potential isomers a) *mer*-1, b) *mer*-2, and c) *fac* in their high-spin state at the M06/TZ2P level. Geometrical similarities between B3LYP/6-31++G(d,p) and M06/TZ2P levels are shown as root mean square deviation (RMSD) in Å.

# Supplementary info

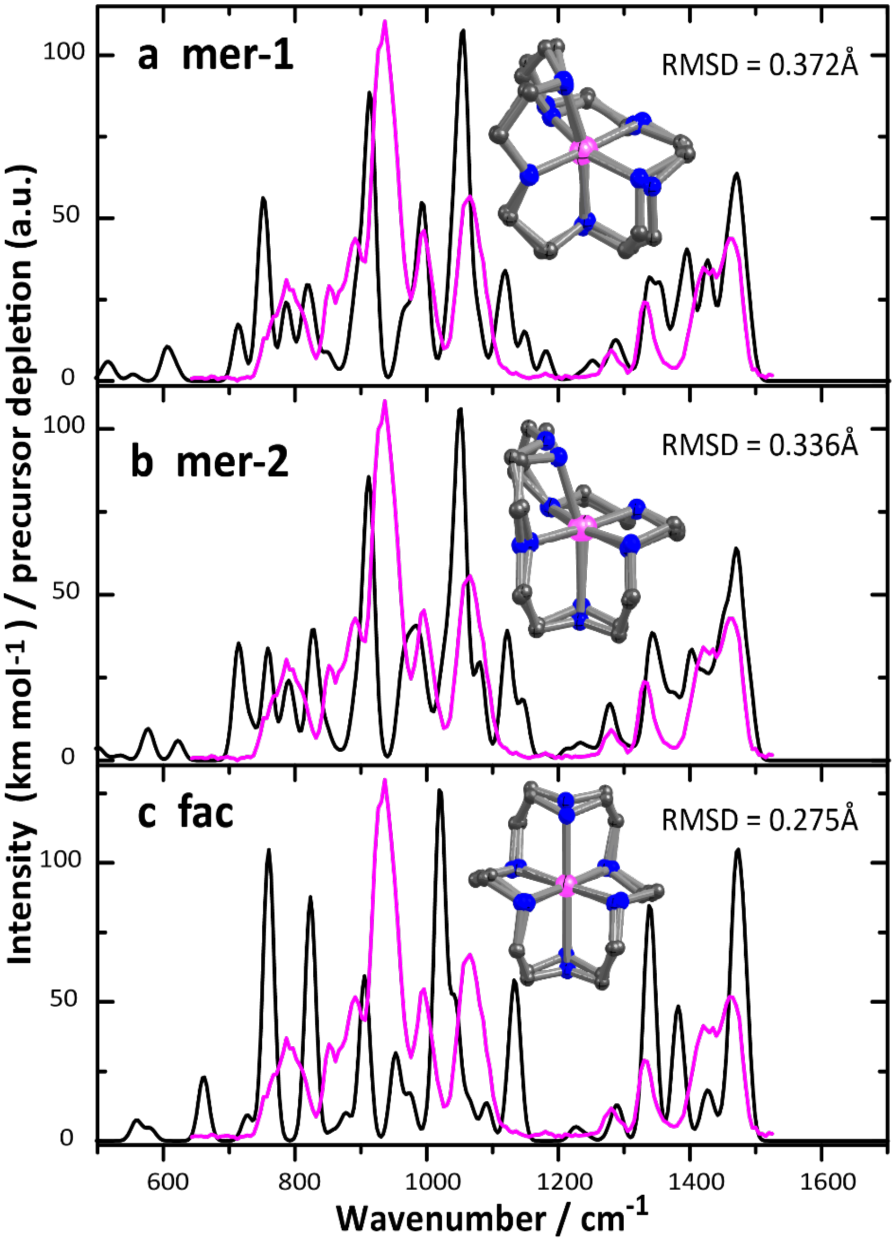


**Figure S3** Experimental IRMPD spectrum (magenta) of $[Ni(hexacyclen)]^{2+}$ overlaid with the calculated IR spectra (black) of the isomers a) *mer*-1, b) *mer*-2, and c) *fac* at the B3LYP/6-31++G(d,p) level in their singlet electronic state. None of the computed spectra agrees with experiment leading us to exclude low-spin isomers. Geometrical similarities between singlet and triplet states are indicated as root mean square deviation (RMSD) in Å.

## Supplementary info

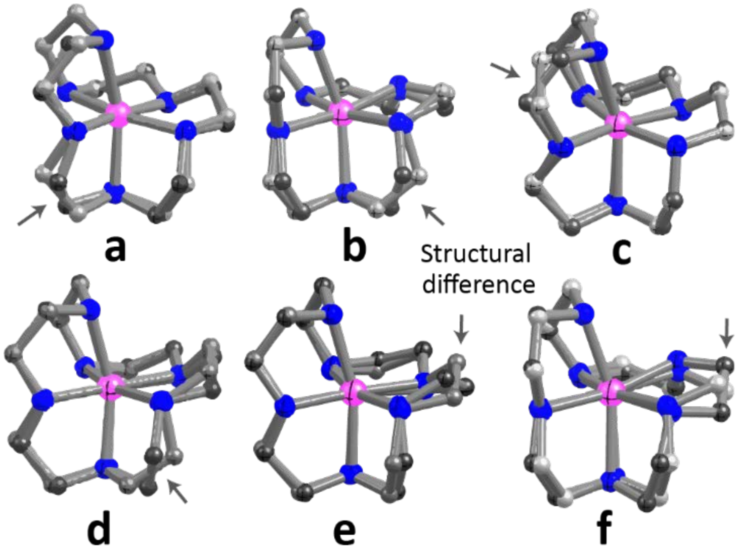


**Figure S4** Six higher energy conformers of *mer-1* are merged individually with the global minimum *mer-1* isomer (a-f). Structural differences are indicated with arrows. H atoms are omitted for clarity. Geometries are calculated in their high-spin state at B3LYP/6-31++G(d,p) level.

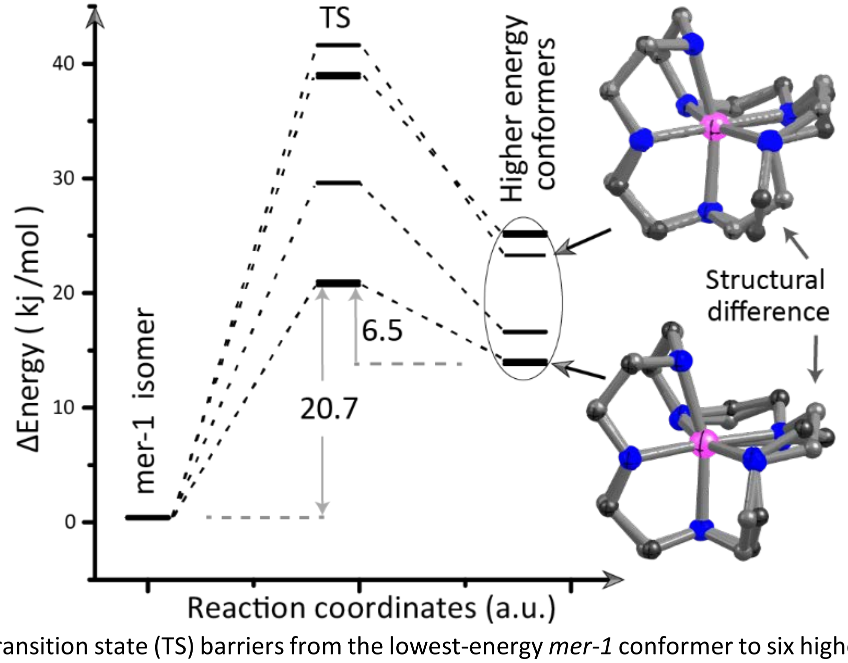


**Figure S5** Computed transition state (TS) barriers from the lowest-energy *mer-1* conformer to six higher energy conformers. Structural differences relative to *mer-1* are indicated with arrows, where *mer-1* is merged with two of the six higher energy conformers. H atoms are omitted for clarity. All geometries are calculated in the high-spin state at B3LYP/6-31++G(d,p) level.

# Supplementary info

## References


(1) Frisch, M. J. , et. al. Gaussian 16, Revision C.01. Gaussian, Inc.: Wallingford CT, **2016**.

(2) Scott, A. P.; Radom, L. Harmonic vibrational frequencies: an evaluation of Hartree-Fock, Møller-Plesset, quadratic configuration interaction, density functional theory, and semiempirical scale factors. *J. Phys. Chem.* **1996**, *100* (41), 16502–16513.

(3) Laury, M. L.; Carlson, M. J.; Wilson, A. K. Vibrational frequency scale factors for density functional theory and the polarization consistent basis sets. *J. Comput. Chem.* **2012**, *33* (30), 2380–2387.

(4) van Lenthe, E.; Baerends, E. J. Optimized Slater-type basis sets for the elements 1–118. *J. Comput. Chem.* **2003**, *24* (9), 1142–1156.

(5) Peverati, R.; Truhlar, D. G. Quest for a universal density functional: the accuracy of density functionals across a broad spectrum of databases in chemistry and physics. *Philos. Trans. R. Soc. A* **2014**, *372* (2011).


## Coordinates (Å) of the optimized geometries at the B3LYP/6-31++G(d,p) level of theory: triplet state isomers

### mer-1

| | | | |
|---|---|---|---|
| C | -1.149989000 | 2.578440000 | -0.930357000 |
| C | -1.595928000 | 1.560554000 | -1.979541000 |
| N | -1.626150000 | 0.171225000 | -1.427411000 |
| C | -2.900011000 | -0.192576000 | -0.741177000 |
| C | -2.899674000 | 0.191472000 | 0.741837000 |
| N | -1.625276000 | -0.171810000 | 1.427354000 |
| N | 0.122392000 | 2.108343000 | -0.334436000 |
| N | 1.727976000 | -0.571584000 | -1.331966000 |
| N | 1.728391000 | 0.572275000 | 1.331705000 |
| C | 0.515244000 | 2.729050000 | 0.951552000 |
| C | 1.811209000 | 2.069243000 | 1.420760000 |
| H | -1.885612000 | 2.678631000 | -0.129856000 |
| H | -1.045465000 | 3.567898000 | -1.390887000 |
| H | -0.884469000 | 1.564578000 | -2.812785000 |
| H | -2.571991000 | 1.843523000 | -2.391379000 |
| H | -3.035449000 | -1.270213000 | -0.853873000 |
| H | -3.759287000 | 0.281101000 | -1.232012000 |
| H | -3.758492000 | -0.282494000 | 1.233191000 |
| H | -3.035430000 | 1.269058000 | 0.854595000 |
| H | -0.295890000 | 2.560640000 | 1.667326000 |
| H | 2.638396000 | 2.407144000 | 0.788666000 |
| H | 2.051397000 | 2.384765000 | 2.440503000 |
| H | 0.655896000 | 3.813902000 | 0.867043000 |
| H | -1.535804000 | -0.456335000 | -2.223650000 |
| H | -1.534646000 | 0.456046000 | 2.223324000 |

# Supplementary info

| | | | |
|---|---|---|---|
| H | 0.863175000 | 2.303082000 | -1.010014000 |
| Ni | 0.062494000 | -0.000081000 | -0.000123000 |
| C | 2.962008000 | 0.044347000 | -0.763767000 |
| H | 2.994695000 | 1.089860000 | -1.086033000 |
| H | 3.867899000 | -0.431404000 | -1.158543000 |
| C | 2.962466000 | -0.042950000 | 0.762820000 |
| H | 2.996066000 | -1.088400000 | 1.085164000 |
| H | 3.868278000 | 0.433420000 | 1.157038000 |
| H | 1.610385000 | -0.210504000 | -2.276522000 |
| C | 1.811649000 | -2.068493000 | -1.421059000 |
| H | 2.639228000 | -2.405896000 | -0.789213000 |
| H | 2.051717000 | -2.383913000 | -2.440818000 |
| H | 1.611517000 | 0.211119000 | 2.276322000 |
| C | 0.516246000 | -2.729102000 | -0.951444000 |
| H | -0.295216000 | -2.561309000 | -1.667013000 |
| H | 0.657587000 | -3.813831000 | -0.866734000 |
| N | 0.123415000 | -2.108316000 | 0.334470000 |
| C | -1.148423000 | -2.579049000 | 0.931014000 |
| H | -1.884365000 | -2.680024000 | 0.130875000 |
| H | -1.043024000 | -3.568257000 | 1.391881000 |
| C | -1.594422000 | -1.560930000 | 1.979995000 |
| H | -2.570265000 | -1.844094000 | 2.392183000 |
| H | -0.882732000 | -1.564470000 | 2.813052000 |
| H | 0.864598000 | -2.302331000 | 1.009812000 |

## mer-2

| | | | |
|---|---|---|---|
| C | -0.539311000 | -2.570697000 | -1.211789000 |
| C | 0.920432000 | -2.366755000 | -1.609527000 |
| N | 1.369349000 | -0.956292000 | -1.357836000 |
| C | 2.751542000 | -0.888879000 | -0.805147000 |
| C | 2.744384000 | -1.133961000 | 0.705770000 |
| N | 1.742652000 | -0.289672000 | 1.422494000 |
| N | -0.727835000 | -2.013057000 | 0.147192000 |
| N | -1.369412000 | 0.956618000 | -1.357185000 |
| N | -1.742556000 | 0.289327000 | 1.422886000 |
| C | -2.106171000 | -2.077256000 | 0.691001000 |
| C | -2.235746000 | -1.062543000 | 1.827119000 |
| H | -0.796603000 | -3.635907000 | -1.266567000 |
| H | -1.213958000 | -2.038083000 | -1.890108000 |
| H | 1.073294000 | -2.642962000 | -2.657409000 |
| H | 1.550559000 | -3.037385000 | -1.016938000 |
| H | 3.160997000 | 0.094496000 | -1.041591000 |
| H | 3.410297000 | -1.624164000 | -1.283687000 |
| H | 3.753639000 | -0.975583000 | 1.105288000 |
| H | 2.490778000 | -2.179224000 | 0.909499000 |
| H | -2.352147000 | -3.080267000 | 1.058697000 |
| H | -3.274319000 | -1.017801000 | 2.176578000 |
| H | -1.625478000 | -1.387327000 | 2.676842000 |
| H | -2.804416000 | -1.861771000 | -0.119755000 |
| H | 1.372937000 | -0.469247000 | -2.251564000 |
| H | 1.511319000 | -0.779399000 | 2.284665000 |
| H | -0.125601000 | -2.539452000 | 0.782110000 |
| Ni | -0.000167000 | 0.000097000 | 0.104028000 |
| C | -2.751616000 | 0.889298000 | -0.804553000 |

## Supplementary info

| | | | |
|---|---|---|---|
| H | -3.161187000 | -0.093922000 | -1.041398000 |
| H | -3.410258000 | 1.624861000 | -1.282838000 |
| C | -2.744457000 | 1.133699000 | 0.706482000 |
| H | -2.491034000 | 2.178914000 | 0.910637000 |
| H | -3.753678000 | 0.974997000 | 1.105959000 |
| H | -1.373021000 | 0.469514000 | -2.250880000 |
| C | -0.920506000 | 2.367033000 | -1.609096000 |
| H | -1.550466000 | 3.037715000 | -1.016421000 |
| H | -1.073561000 | 2.643226000 | -2.656929000 |
| H | -1.511218000 | 0.778920000 | 2.285134000 |
| C | 0.539325000 | 2.570844000 | -1.211657000 |
| H | 1.213811000 | 2.038280000 | -1.890165000 |
| H | 0.796661000 | 3.636061000 | -1.266385000 |
| N | 0.728012000 | 2.013051000 | 0.147240000 |
| C | 2.106456000 | 2.076862000 | 0.690782000 |
| H | 2.804458000 | 1.861218000 | -0.120147000 |
| H | 2.352773000 | 3.079801000 | 1.058447000 |
| C | 2.236015000 | 1.062087000 | 1.826850000 |
| H | 3.274623000 | 1.017146000 | 2.176193000 |
| H | 1.625881000 | 1.386896000 | 2.676663000 |
| H | 0.126047000 | 2.539516000 | 0.782349000 |

## fac

| | | | |
|---|---|---|---|
| C | -1.584935000 | -2.382964000 | 0.887901000 |
| C | -1.200416000 | -2.754542000 | -0.544553000 |
| N | -0.064668000 | -1.926965000 | -1.053758000 |
| C | 1.271250000 | -2.563341000 | -0.888347000 |
| C | 1.786020000 | -2.416928000 | 0.543963000 |
| N | 1.637488000 | -1.019872000 | 1.054086000 |
| N | -1.701762000 | -0.907951000 | 1.054056000 |
| N | -1.637627000 | 1.019781000 | -1.054028000 |
| N | 0.064655000 | 1.927018000 | 1.053673000 |
| C | -2.986116000 | -0.337924000 | 0.544539000 |
| C | -2.856507000 | 0.180901000 | -0.887963000 |
| H | -0.829753000 | -2.730989000 | 1.595982000 |
| H | -2.522789000 | -2.886872000 | 1.152623000 |
| H | -2.052269000 | -2.604382000 | -1.211359000 |
| H | -0.956775000 | -3.822495000 | -0.591158000 |
| H | 1.949898000 | -2.083029000 | -1.596568000 |
| H | 1.238647000 | -3.627380000 | -1.153541000 |
| H | 2.832749000 | -2.739932000 | 0.589756000 |
| H | 1.230489000 | -3.080001000 | 1.210737000 |
| H | -3.282066000 | 0.474978000 | 1.211221000 |
| H | -2.780331000 | -0.646954000 | -1.596295000 |
| H | -3.761910000 | 0.741077000 | -1.152602000 |
| H | -3.789316000 | -1.082758000 | 0.591066000 |
| H | -0.207689000 | -1.805284000 | -2.054351000 |
| H | 1.461285000 | -1.083441000 | 2.054768000 |
| H | -1.668288000 | -0.723621000 | 2.054737000 |
| Ni | -0.000031000 | -0.000003000 | 0.000043000 |
| C | -1.786102000 | 2.416906000 | -0.544039000 |
| H | -2.832838000 | 2.739897000 | -0.589735000 |
| H | -1.230668000 | 3.079898000 | -1.210979000 |

# Supplementary info

| | | | |
|---|---|---|---|
| C | -1.271164000 | 2.563547000 | 0.888204000 |
| H | -1.238388000 | 3.627645000 | 1.153144000 |
| H | -1.949836000 | 2.083519000 | 1.596593000 |
| H | -1.461477000 | 1.083284000 | -2.054725000 |
| C | 1.200536000 | 2.754448000 | 0.544437000 |
| H | 0.957029000 | 3.822427000 | 0.591100000 |
| H | 2.052379000 | 2.604144000 | 1.211226000 |
| H | 0.207666000 | 1.805422000 | 2.054277000 |
| C | 1.584956000 | 2.382914000 | -0.888038000 |
| H | 0.829665000 | 2.730854000 | -1.596047000 |
| H | 2.522745000 | 2.886891000 | -1.152846000 |
| N | 1.701905000 | 0.907897000 | -1.054169000 |
| C | 2.986223000 | 0.337960000 | -0.544301000 |
| H | 3.282500000 | -0.474793000 | -1.211023000 |
| H | 3.789303000 | 1.082941000 | -0.590514000 |
| C | 2.856327000 | -0.180940000 | 0.888160000 |
| H | 3.761709000 | -0.741094000 | 1.152920000 |
| H | 2.780027000 | 0.646921000 | 1.596475000 |
| H | 1.668808000 | 0.723623000 | -2.054874000 |

## 2. Higher energy conformers (total 6) of mer-1 isomer (see Figure S3)

Excited-1

| | | | |
|---|---|---|---|
| C | 1.550656000 | 2.465350000 | 0.721954000 |
| C | 1.899655000 | 1.463143000 | 1.824021000 |
| N | 1.702806000 | 0.047029000 | 1.381362000 |
| C | 2.895134000 | -0.554359000 | 0.718124000 |
| C | 2.921846000 | -0.266116000 | -0.782084000 |
| N | 1.608369000 | -0.556984000 | -1.422116000 |
| N | 0.195336000 | 2.142650000 | 0.223298000 |
| N | -1.645117000 | -0.180358000 | 1.410277000 |
| N | -1.661201000 | 0.753435000 | -1.314650000 |
| C | -0.228316000 | 2.775017000 | -1.047366000 |
| C | -1.625488000 | 2.251361000 | -1.379099000 |
| H | 2.247764000 | 2.407259000 | -0.116251000 |
| H | 1.611005000 | 3.486768000 | 1.116604000 |
| H | 1.240686000 | 1.631479000 | 2.683192000 |
| H | 2.925550000 | 1.627577000 | 2.174091000 |
| H | 2.863093000 | -1.632256000 | 0.894229000 |
| H | 3.824711000 | -0.190841000 | 1.173229000 |
| H | 3.724351000 | -0.852741000 | -1.246420000 |
| H | 3.156052000 | 0.783791000 | -0.969030000 |
| H | 0.492836000 | 2.495797000 | -1.822885000 |
| H | -2.342453000 | 2.645717000 | -0.651991000 |
| H | -1.948415000 | 2.609140000 | -2.361547000 |
| H | -0.239647000 | 3.870397000 | -0.986785000 |
| H | 1.533163000 | -0.498076000 | 2.224068000 |
| H | 1.591338000 | -0.040507000 | -2.299079000 |
| H | -0.466270000 | 2.437855000 | 0.943423000 |
| Ni | -0.043464000 | 0.039959000 | -0.020677000 |
| C | -2.924549000 | 0.288173000 | 0.794414000 |
| H | -3.067878000 | 1.327775000 | 1.103677000 |
| H | -3.782731000 | -0.270729000 | 1.186460000 |

# Supplementary info

| | | | |
|---|---|---|---|
| C | -2.922330000 | 0.203587000 | -0.738196000 |
| H | -3.015159000 | -0.833729000 | -1.070221000 |
| H | -3.804120000 | 0.730625000 | -1.123250000 |
| H | -1.502648000 | 0.374448000 | 2.251778000 |
| C | -1.601395000 | -1.606322000 | 1.849117000 |
| H | -2.480507000 | -1.865884000 | 2.451815000 |
| H | -0.725382000 | -1.703414000 | 2.498167000 |
| H | -1.580680000 | 0.404071000 | -2.267907000 |
| C | -1.483031000 | -2.570022000 | 0.662854000 |
| H | -1.122459000 | -3.540823000 | 1.016417000 |
| H | -2.465677000 | -2.750322000 | 0.218630000 |
| N | -0.576707000 | -2.016586000 | -0.386538000 |
| C | 0.663748000 | -2.768932000 | -0.681165000 |
| H | 1.243971000 | -2.856821000 | 0.240328000 |
| H | 0.452870000 | -3.786833000 | -1.032208000 |
| C | 1.417217000 | -2.001821000 | -1.768944000 |
| H | 2.378324000 | -2.485313000 | -1.977835000 |
| H | 0.835378000 | -2.044358000 | -2.696750000 |
| H | -1.103367000 | -1.997572000 | -1.258201000 |

Excited-2

| | | | |
|---|---|---|---|
| C | -0.662343000 | 2.769426000 | -0.680207000 |
| C | -1.416591000 | 2.003134000 | -1.768023000 |
| N | -1.607948000 | 0.558056000 | -1.422099000 |
| C | -2.921491000 | 0.267077000 | -0.782323000 |
| C | -2.894830000 | 0.555343000 | 0.717871000 |
| N | -1.702669000 | -0.046321000 | 1.381263000 |
| N | 0.578148000 | 2.016822000 | -0.386812000 |
| N | 1.660864000 | -0.754113000 | -1.314694000 |
| N | 1.644860000 | 0.179524000 | 1.410472000 |
| C | 1.485127000 | 2.568921000 | 0.662417000 |
| C | 1.601713000 | 1.605498000 | 1.849064000 |
| H | -1.241975000 | 2.856854000 | 0.241709000 |
| H | -0.451646000 | 3.787541000 | -1.030801000 |
| H | -0.835297000 | 2.046250000 | -2.696135000 |
| H | -2.377730000 | 2.486958000 | -1.975990000 |
| H | -3.155554000 | -0.782847000 | -0.969308000 |
| H | -3.723965000 | 0.853678000 | -1.246740000 |
| H | -3.824519000 | 0.192023000 | 1.172895000 |
| H | -2.862629000 | 1.633252000 | 0.893916000 |
| H | 1.126492000 | 3.540540000 | 1.015785000 |
| H | 2.480453000 | 1.864680000 | 2.452479000 |
| H | 0.725087000 | 1.703308000 | 2.497183000 |
| H | 2.468195000 | 2.747237000 | 0.218291000 |
| H | -1.590957000 | 0.042113000 | -2.299373000 |
| H | -1.532833000 | 0.498901000 | 2.223857000 |
| H | 1.104262000 | 1.997880000 | -1.258794000 |
| Ni | 0.043393000 | -0.039958000 | -0.021029000 |
| C | 2.922216000 | -0.205226000 | -0.737964000 |
| H | 3.016055000 | 0.831987000 | -1.069986000 |
| H | 3.803673000 | -0.733016000 | -1.122796000 |
| C | 2.924154000 | -0.289602000 | 0.794737000 |
| H | 3.067211000 | -1.329148000 | 1.104307000 |
| H | 3.782441000 | 0.269221000 | 1.186653000 |

# Supplementary info

| | | | |
|---|---|---|---|
| H | 1.580756000 | -0.404673000 | -2.267959000 |
| C | 1.624184000 | -2.251972000 | -1.379116000 |
| H | 2.340663000 | -2.646698000 | -0.651722000 |
| H | 1.947257000 | -2.610081000 | -2.361434000 |
| H | 1.501992000 | -0.375189000 | 2.251964000 |
| C | 0.226648000 | -2.774909000 | -1.047771000 |
| H | -0.494269000 | -2.494934000 | -1.823268000 |
| H | 0.237365000 | -3.870339000 | -0.987721000 |
| N | -0.196638000 | -2.142812000 | 0.223085000 |
| C | -1.551865000 | -2.464862000 | 0.722223000 |
| H | -2.249306000 | -2.406738000 | -0.115699000 |
| H | -1.612539000 | -3.486156000 | 1.117159000 |
| C | -1.900023000 | -1.462284000 | 1.824248000 |
| H | -2.925834000 | -1.626169000 | 2.174807000 |
| H | -1.240722000 | -1.630751000 | 2.683140000 |
| H | 0.465155000 | -2.438208000 | 0.942951000 |

Excited-3

| | | | |
|---|---|---|---|
| C | 0.919526000 | -2.825107000 | -0.749976000 |
| C | 1.436773000 | -1.914432000 | -1.864208000 |
| N | 1.728885000 | -0.559782000 | -1.311458000 |
| C | 2.984638000 | -0.567562000 | -0.502982000 |
| C | 2.984468000 | 0.568753000 | 0.502739000 |
| N | 1.728770000 | 0.560464000 | 1.311393000 |
| N | -0.211243000 | -2.143945000 | -0.076541000 |
| N | -1.664436000 | 0.343848000 | -1.418072000 |
| N | -1.664360000 | -0.344419000 | 1.418111000 |
| C | -0.517842000 | -2.573183000 | 1.306613000 |
| C | -1.753748000 | -1.802957000 | 1.772697000 |
| H | 1.692898000 | -3.024065000 | -0.004175000 |
| H | 0.623765000 | -3.796814000 | -1.161061000 |
| H | 0.677241000 | -1.804833000 | -2.645688000 |
| H | 2.323105000 | -2.357354000 | -2.334852000 |
| H | 3.869069000 | -0.504126000 | -1.147324000 |
| H | 3.048660000 | -1.521399000 | 0.024407000 |
| H | 3.047927000 | 1.522580000 | -0.024709000 |
| H | 3.868996000 | 0.505713000 | 1.147047000 |
| H | 0.352559000 | -2.344808000 | 1.930468000 |
| H | -2.644177000 | -2.222200000 | 1.292980000 |
| H | -1.894267000 | -1.928029000 | 2.849999000 |
| H | -0.702393000 | -3.652383000 | 1.380809000 |
| H | 1.867853000 | 0.073163000 | -2.098480000 |
| H | 1.868163000 | -0.072465000 | 2.098353000 |
| H | -1.042334000 | -2.321743000 | -0.640979000 |
| Ni | 0.008343000 | -0.000057000 | -0.000006000 |
| C | -2.894877000 | -0.168210000 | -0.746096000 |
| H | -2.935520000 | -1.252484000 | -0.891156000 |
| H | -3.802544000 | 0.236410000 | -1.209991000 |
| C | -2.895051000 | 0.166938000 | 0.746131000 |
| H | -2.936376000 | 1.251178000 | 0.891234000 |
| H | -3.802514000 | -0.238259000 | 1.209934000 |
| H | -1.547928000 | -0.177462000 | -2.284363000 |
| C | -1.754598000 | 1.802354000 | -1.772747000 |
| H | -2.645455000 | 2.221111000 | -1.293416000 |
| H | -1.894715000 | 1.927218000 | -2.850123000 |
| H | -1.548224000 | 0.176879000 | 2.284460000 |

# Supplementary info

| | | | |
|---|---|---|---|
| C | -0.519206000 | 2.573278000 | -1.306353000 |
| H | 0.351346000 | 2.345813000 | -1.930340000 |
| H | -0.704647000 | 3.652349000 | -1.380194000 |
| N | -0.212162000 | 2.143832000 | 0.076586000 |
| C | 0.918376000 | 2.825412000 | 0.750004000 |
| H | 1.691596000 | 3.024639000 | 0.004122000 |
| H | 0.622317000 | 3.797030000 | 1.161091000 |
| C | 1.436186000 | 1.914989000 | 1.864205000 |
| H | 2.322380000 | 2.358265000 | 2.334780000 |
| H | 0.676781000 | 1.805067000 | 2.645771000 |
| H | -1.043215000 | 2.320994000 | 0.641282000 |

Excited-4

| | | | |
|---|---|---|---|
| C | -1.098314000 | 2.421390000 | -1.310147000 |
| C | -1.481781000 | 1.244361000 | -2.204806000 |
| N | -1.537670000 | -0.037596000 | -1.438706000 |
| C | -2.817489000 | -0.286462000 | -0.711728000 |
| C | -2.817609000 | 0.285816000 | 0.711785000 |
| N | -1.537696000 | 0.037384000 | 1.438822000 |
| N | 0.132530000 | 2.076614000 | -0.553263000 |
| N | 1.769844000 | -0.840837000 | -1.193469000 |
| N | 1.769779000 | 0.841245000 | 1.193351000 |
| C | 0.332845000 | 2.854618000 | 0.693729000 |
| C | 1.509465000 | 2.281459000 | 1.517038000 |
| H | -1.889264000 | 2.642465000 | -0.590484000 |
| H | -0.964897000 | 3.323967000 | -1.917432000 |
| H | -0.727574000 | 1.118609000 | -2.989205000 |
| H | -2.436010000 | 1.448409000 | -2.705559000 |
| H | -2.967672000 | -1.367788000 | -0.683328000 |
| H | -3.669634000 | 0.130598000 | -1.262619000 |
| H | -3.669599000 | -0.131574000 | 1.262648000 |
| H | -2.968185000 | 1.367090000 | 0.683377000 |
| H | -0.598859000 | 2.783654000 | 1.261799000 |
| H | 2.420719000 | 2.862157000 | 1.342462000 |
| H | 1.282758000 | 2.377771000 | 2.581670000 |
| H | 0.497915000 | 3.919297000 | 0.491107000 |
| H | -1.444255000 | -0.782296000 | -2.125719000 |
| H | -1.444567000 | 0.782116000 | 2.125842000 |
| H | 0.927592000 | 2.245843000 | -1.170156000 |
| Ni | 0.115051000 | 0.000078000 | 0.000019000 |
| C | 2.984203000 | -0.679286000 | -0.338332000 |
| H | 3.902082000 | -0.814778000 | -0.922828000 |
| H | 2.976721000 | -1.471759000 | 0.417368000 |
| C | 2.984288000 | 0.679272000 | 0.338418000 |
| H | 3.902079000 | 0.814735000 | 0.923039000 |
| H | 2.976965000 | 1.471607000 | -0.417434000 |
| H | 1.946229000 | -0.350740000 | -2.068945000 |
| C | 1.509272000 | -2.280854000 | -1.517691000 |
| H | 2.420712000 | -2.861648000 | -1.344379000 |
| H | 1.281555000 | -2.376439000 | -2.582171000 |
| H | 1.945907000 | 0.351291000 | 2.068955000 |
| C | 0.333405000 | -2.854657000 | -0.693615000 |
| H | -0.598613000 | -2.784492000 | -1.261266000 |
| H | 0.499330000 | -3.919165000 | -0.490818000 |
| N | 0.133080000 | -2.076574000 | 0.553342000 |
| C | -1.097702000 | -2.421543000 | 1.310310000 |

# Supplementary info

| | | | |
|---|---|---|---|
| H | -1.888628000 | -2.642791000 | 0.590682000 |
| H | -0.964078000 | -3.324094000 | 1.917590000 |
| C | -1.481375000 | -1.244559000 | 2.204943000 |
| H | -2.435497000 | -1.448812000 | 2.705806000 |
| H | -0.727116000 | -1.118581000 | 2.989261000 |
| H | 0.928143000 | -2.245826000 | 1.170227000 |

Excited-5

| | | | |
|---|---|---|---|
| C | 0.204165000 | -2.814243000 | -1.261250000 |
| C | 1.611974000 | -2.244161000 | -1.181075000 |
| N | 1.591305000 | -0.755313000 | -1.308826000 |
| C | 2.878882000 | -0.158948000 | -0.865581000 |
| C | 3.018375000 | -0.258114000 | 0.658199000 |
| N | 1.762825000 | 0.121102000 | 1.373426000 |
| N | -0.701696000 | -2.064100000 | -0.353693000 |
| N | -1.688512000 | 0.720024000 | -1.331301000 |
| N | -1.644471000 | -0.198371000 | 1.437858000 |
| C | -0.885025000 | -2.560701000 | 1.029533000 |
| C | -1.926149000 | -1.654547000 | 1.696682000 |
| H | 0.215263000 | -3.886828000 | -1.034853000 |
| H | -0.188951000 | -2.704972000 | -2.277446000 |
| H | 2.251864000 | -2.696389000 | -1.947860000 |
| H | 2.050853000 | -2.494013000 | -0.213409000 |
| H | 2.895524000 | 0.879952000 | -1.201316000 |
| H | 3.735308000 | -0.659711000 | -1.335260000 |
| H | 3.858293000 | 0.367014000 | 0.983139000 |
| H | 3.273122000 | -1.280324000 | 0.946794000 |
| H | 0.076270000 | -2.513723000 | 1.550641000 |
| H | -2.918908000 | -1.892701000 | 1.299947000 |
| H | -1.966191000 | -1.848906000 | 2.771873000 |
| H | -1.226471000 | -3.603356000 | 1.057320000 |
| H | 1.467649000 | -0.529012000 | -2.295963000 |
| H | 1.691069000 | -0.479285000 | 2.192032000 |
| H | -1.622628000 | -2.069158000 | -0.787532000 |
| Ni | -0.051334000 | -0.022500000 | -0.048857000 |
| C | -2.953949000 | 0.375585000 | -0.617384000 |
| H | -3.206412000 | -0.662812000 | -0.853100000 |
| H | -3.794165000 | 0.981583000 | -0.977296000 |
| C | -2.812579000 | 0.557928000 | 0.895509000 |
| H | -2.663928000 | 1.615802000 | 1.135208000 |
| H | -3.746816000 | 0.259231000 | 1.386084000 |
| H | -1.706416000 | 0.251647000 | -2.234805000 |
| C | -1.529228000 | 2.192503000 | -1.581999000 |
| H | -2.270530000 | 2.728683000 | -0.980280000 |
| H | -1.744331000 | 2.432270000 | -2.627182000 |
| H | -1.393600000 | 0.228653000 | 2.326646000 |
| C | -0.128074000 | 2.669810000 | -1.200951000 |
| H | 0.622946000 | 2.307150000 | -1.910592000 |
| H | -0.090182000 | 3.766416000 | -1.210717000 |
| N | 0.188139000 | 2.105280000 | 0.130628000 |
| C | 1.476403000 | 2.505305000 | 0.741144000 |
| H | 2.252192000 | 2.478833000 | -0.027041000 |
| H | 1.443655000 | 3.531661000 | 1.124997000 |
| C | 1.776850000 | 1.526264000 | 1.872073000 |
| H | 2.736348000 | 1.771661000 | 2.343198000 |
| H | 1.007779000 | 1.613887000 | 2.648185000 |

# Supplementary info

| | | | |
|---|---|---|---|
| H | -0.544255000 | 2.416319000 | 0.770564000 |

Excited-6

| | | | |
|---|---|---|---|
| C | 1.476389000 | 2.505389000 | 0.741222000 |
| C | 1.776472000 | 1.526142000 | 1.872068000 |
| N | 1.762104000 | 0.120968000 | 1.373343000 |
| C | 3.017709000 | -0.258850000 | 0.658658000 |
| C | 2.878881000 | -0.159026000 | -0.865082000 |
| N | 1.591187000 | -0.754956000 | -1.308821000 |
| N | 0.188028000 | 2.105985000 | 0.130681000 |
| N | -1.644370000 | -0.198280000 | 1.437593000 |
| N | -1.687932000 | 0.719841000 | -1.331207000 |
| C | -0.127928000 | 2.670102000 | -1.201010000 |
| C | -1.528716000 | 2.192228000 | -1.582672000 |
| H | 2.252194000 | 2.478542000 | -0.026958000 |
| H | 1.444265000 | 3.531762000 | 1.125117000 |
| H | 1.007357000 | 1.613915000 | 2.648131000 |
| H | 2.735994000 | 1.771179000 | 2.343396000 |
| H | 3.271647000 | -1.281387000 | 0.946866000 |
| H | 3.857925000 | 0.365540000 | 0.984238000 |
| H | 3.735239000 | -0.659963000 | -1.334659000 |
| H | 2.895884000 | 0.879959000 | -1.200526000 |
| H | 0.623521000 | 2.307662000 | -1.910339000 |
| H | -2.270567000 | 2.728569000 | -0.981776000 |
| H | -1.743186000 | 2.431335000 | -2.628146000 |
| H | -0.090447000 | 3.766735000 | -1.211033000 |
| H | 1.689481000 | -0.479477000 | 2.191848000 |
| H | 1.467522000 | -0.527956000 | -2.295784000 |
| H | -0.544378000 | 2.417077000 | 0.770585000 |
| Ni | -0.051161000 | -0.022377000 | -0.048474000 |
| C | -2.812436000 | 0.558157000 | 0.895301000 |
| H | -2.663714000 | 1.616011000 | 1.135032000 |
| H | -3.746720000 | 0.259496000 | 1.385815000 |
| C | -2.953688000 | 0.375760000 | -0.617552000 |
| H | -3.206325000 | -0.662610000 | -0.853233000 |
| H | -3.793674000 | 0.981949000 | -0.977665000 |
| H | -1.393424000 | 0.228758000 | 2.326349000 |
| C | -1.926265000 | -1.654436000 | 1.696486000 |
| H | -2.919018000 | -1.892428000 | 1.299655000 |
| H | -1.966423000 | -1.848756000 | 2.771682000 |
| H | -1.705470000 | 0.250934000 | -2.234446000 |
| C | -0.885277000 | -2.560812000 | 1.029500000 |
| H | 0.076022000 | -2.513854000 | 1.550584000 |
| H | -1.226899000 | -3.603411000 | 1.057573000 |
| N | -0.701780000 | -2.064626000 | -0.353815000 |
| C | 0.204263000 | -2.814684000 | -1.261088000 |
| H | 0.216442000 | -3.887097000 | -1.033634000 |
| H | -0.189499000 | -2.706924000 | -2.277204000 |
| C | 1.611882000 | -2.243745000 | -1.181942000 |
| H | 2.051633000 | -2.494134000 | -0.214726000 |
| H | 2.251317000 | -2.695624000 | -1.949393000 |
| H | -1.622570000 | -2.069109000 | -0.787844000 |
